# Label-free quantitative proteomics in *Candida* yeast species: technical and biological replicates to assess data reproducibility

**DOI:** 10.1186/s13104-019-4505-8

**Published:** 2019-08-01

**Authors:** Gaëlle Lelandais, Thomas Denecker, Camille Garcia, Nicolas Danila, Thibaut Léger, Jean-Michel Camadro

**Affiliations:** 1 CEA, CNRS, Institute for Integrative Biology of the Cell (I2BC), Univ. Paris-Sud, Gif-Sur-Yvette, France; 2 Mass Spectrometry Laboratory, CNRS, Institut Jacques Monod, UMR 7592, Université de Paris, 75205 Paris, France; 3 CNRS, Institut Jacques Monod (IJM), Univ. Paris Diderot, Paris, France

**Keywords:** Mass spectrometry, Label-free quantitative proteomics, *Candida glabrata*, *Candida albicans*, Alkaline pH

## Abstract

**Objective:**

Label-free quantitative proteomics has emerged as a powerful strategy to obtain high quality quantitative measures of the proteome with only a very small quantity of total protein extract. Because our research projects were requiring the application of bottom-up shotgun mass spectrometry proteomics in the pathogenic yeasts *Candida glabrata* and *Candida albicans*, we performed preliminary experiments to (i) obtain a precise list of all the proteins for which measures of abundance could be obtained and (ii) assess the reproducibility of the results arising respectively from biological and technical replicates.

**Data description:**

Three time-courses were performed in each *Candida* species, and an alkaline pH stress was induced for two of them. Cells were collected 10 and 60 min after stress induction and proteins were extracted. Samples were analysed two times by mass spectrometry. Our final dataset thus comprises label-free quantitative proteomics results for 24 samples (two species, three time-courses, two time points and two runs of mass spectrometry). Statistical procedures were applied to identify proteins with differential abundances between stressed and unstressed situations. Considering that *C. glabrata* and *C. albicans* are human pathogens, which face important pH fluctuations during a human host infection, this dataset has a potential value to other researchers in the field.

## Objective

Studying proteome dynamics is a key step in systems biology projects. In this context, label-free bottom-up shotgun MS-based proteomics produces quantitative analyses of proteomes. This technique has emerged from significant improvements achieved by mass spectrometry (MS) instrumentation, chromatographic separation systems and a stronger correlation between the relative measured ion intensity and the original molecule abundance in the electrospray ionization process [1–3]. Members of our research team were involved in functional genomics studies in pathogenic yeasts *Candida glabrata* and *Candida albicans* [4–8]. We observed how the experimental design is a critical step to empower the statistics used to assess the robustness of the results.

“How many replicates is enough?” is certainly one of the most frequently asked questions in wet laboratories. This question is especially critical in situations where the experiments are expensive, and/or the preparation of the biological samples is challenging. Here, our objective was to assess the robustness of the results arising from label-free bottom-up shotgun MS-based proteomics performed in *C. glabrata* and *C. albicans*, in case of technical and biological replicates. If the importance of biological replicates was indisputable when we started this project, the interest for technical replicates was more questionable.

We induced proteome modifications applying an alkaline pH stress to *Candida* cells grown in minimal liquid medium. Our final dataset comprises quantitative proteomics for 24 samples (two species, three time-courses, two time points and two runs of mass spectrometry, see below) [9, 10]. We believe it could be useful for other researchers, either interested in a statistical exploitation of the results (to model for instance the variability of protein quantifications associated with biological or technical replicates respectively) or interested in a better understanding of the cellular mechanisms which underly adaptation of pathogenic yeasts to pH changes, a key process during a human host infection [11].

## Data description

In this analysis, we performed in *Candida glabrata* (CGLAB) and *Candida albicans* (CALB) yeast species, three cultures referred as CTRL, ALK1 and ALK2. CGLAB and CALB strains are respectively the ones used in [4] and [7], and they were cultured in the same standard conditions as described in [4, 7]. Here, CTRL means “Control”, i.e. the cells were grown in minimal liquid medium. ALK means “alkaline pH stress”, i.e. the cells were subjected to an alkaline stress by adding 1 M of Tris base. This dose was appropriate to slightly affect cell growth without killing the cells. ALK1 and ALK2 referred to two biological replicates, i.e. independent cell growth cultures. T10 and T60 means respectively “time point 10 min” and “time point 60 min”, i.e. the time after stress induction at which the cells were collected for mass spectrometry experiments. These time points were chosen because the cells were then in the exponential phase. Finally, REP1 and REP2 referred to two technical replicates, i.e. independent MS acquisition from the same protein extract and trypsin digestion.

Overall, two datasets were associated to this paper note (Table 1). Data set 1 comprises 24 raw data files, obtained from a Q-Exactive Plus mass spectrometer coupled to a Nano_LC Proseon 1000 equipped with an easy spray ion source (all from Thermo Fisher Scientific); 48 search files, obtained with the Proteome Discovered software (Thermo Scientific, version 2.1) and the Mascot search engine (Matrix Science, version 2.5.1); 2 quantification files obtained with the Progenesis QI for Proteomics software (version 4.1, Waters) and 2 FASTA files obtained for the CGD website and used for the MS/MS identification step. Note that detailed descriptions of (i) sample processing protocol and (ii) data processing protocol can be found in [9]. Data file 2 explains the relationship between MS files and associated experimental conditions (CTRL, ALK1, ALK2, T10, T60, REP1 and REP2).

**Table 1:** Overview of the data files related to the study of label-free quantitative proteomics in *Candida* yeasts species, assessing data reproducibility in technical and biological replicates

Label	Name of data file	File types	Data repository and identifier (accession number)
Data set 1 [9]	Proteomics project in *Candida* yeast species	RAW files (.raw), Search files from Proteome Discoverer software (.pdResults and.xlsx), Quantification files from Progenesis QI for Proteomics software (.csv), FASTA files (.fasta)	http://identifiers.org/pride.project:PXD014125
Data file 2 [10]	Detailed correspondence between MS files and experimental conditions	PDF (.pdf)	10.5281/zenodo.3334949

## Limitations

We produced this dataset to assess our ability to properly quantify protein abundances in yeasts *Candida glabrata* and *Candida albicans*. An open question for us was the impact of technical replicates compared to biological replicates. We thus performed cell cultures under two different conditions (control and induced stress), collected cells at two separate time points (10 and 60 min) after stress induction, extracted the proteins, performed trypsin digestions and analysed the composition of samples by mass spectrometry. As a result, we were first able to observe a good coverage of proteome in yeasts *C. glabrata* and *C. albicans*, respectively. Between 1500 and 2000 proteins were identified in a reproducible way, representing ~ 30% of the total protein repositories in these species. It should be noted that a problem in two sample preparations occurred in *Candida glabrata*. Less than 250 proteins were found in technical replicates 1445007-Q3 and 1445007-Q9, which are CGLAB, ALK2, T10, REP1 and REP2 [10]. This is the main limitation for our data. Second, we observed that technical replicates were critical to increase the number of identified proteins, as ~ 25% of them were found in only one technical replicate. In this context, having a third technical replicate would have been of interest to see if better proteome coverage can still be obtained. Finally, we were able to observe very high positive correlation values (higher than 0.9) between abundances of proteins obtained from biological replicates. If this result is very encouraging, it may also reflect that our cell cultures were not totally “independent”. Indeed, they were performed simultaneously, starting from the same over-night pre-culture. We believe it could be interesting to replicate these experiments paying more attention to this last point, in the design of experiments.

## Data Availability

The mass spectrometry proteomics data have been deposited to the ProteomeXchange Consortium via the PRIDE partner repository [10] with the dataset identifier PXD014125 [9]. Please see Table 1 for details and links to the data.

## Abbreviations

*C. glabrata* and CGLAB: *Candida glabrata*
*C. albicans* and CALB: *Candida albicans*
MS: mass spectrometry
CTLR: control
ALK1 and ALK2: alkaline stress 1 and 2
T10 and T60: time point 10 min and time point 60 min
REP1 and REP2: replicate 1 and replicate 2
